# Electronic Cigarette Use and Its Relationship with Smoking and Alcohol and Illicit Drug Consumption among Romanian University Students

**DOI:** 10.3390/medicina57020137

**Published:** 2021-02-04

**Authors:** Lucia Maria Lotrean, Milena Man, Cristina Gavrilescu, Mira Florea

**Affiliations:** 1 Department of Community Medicine, Iuliu Hatieganu University of Medicine and Pharmacy, 400012 Cluj-Napoca, Romania; llotrean@umfcluj.ro (L.M.L.); miraflorea@umfcluj.ro (M.F.); 2 Department of Medical Specialties, Iuliu Hatieganu University of Medicine and Pharmacy, 400012 Cluj-Napoca, Romania; manmilena50@yahoo.com; 3 First Medical Department, Gr. T. Popa, University of Medicine and Pharmacy, 700115 Iasi, Romania

**Keywords:** e-cigarettes, youth, health risk behaviors, Romania

## Abstract

*Background and objectives:* This study assesses electronic cigarette (e-cigarette) use and its relationship with smoking and alcohol and illicit drug consumption among Romanian university students. *Materials and methods:* A cross sectional study using anonymous questionnaire was performed in 2017 among 400 university students from Cluj-Napoca, Romania. *Results*: 95.5% of the participants had heard about e-cigarettes and 43.7% of these had tried e-cigarettes during their lifetime, while 8.9% declared using cigarettes in the previous month (one out of five students who had tried them during their lifetime). Half of the students had smoked during their lifetime and one third had smoked in the previous month. Eighty-five percent of participants had experimented alcohol intoxication during their lifetime and 45% had done so in the previous month, while illicit drug use during their lifetime and the previous month was 34% and 9.5%, respectively. The results of the linear regression analyses show a positive correlation between e-cigarette use, smoking, experimentation with alcohol intoxication, and the use of illicit drugs. *Conclusions*: Future studies as well as educational activities should address the complex relationship between e-cigarette and other substance use among Romanian youth.

## 1. Introduction

The use of electronic cigarettes (e-cigarettes) among youth has grown exponentially in the past few years in several countries, their popularity being attributed to a variety of factors such as successful marketing targeting youth and the attraction derived from their novelty, product design, and flavor availability [1,2,3,4,5,6,7].

Their use might lead to several health problems such as acute toxicity, asthma, adverse brain development, adverse fetal development, lung cancer, and injuries related to e-cigarette battery explosions [4,8,9]. Moreover, even though they are promoted as aids for quitting smoking, several studies show that such advantages are less relevant to youth, while young people using e-cigarettes are more likely to later initiate combustible cigarette use or to continue dual use of both traditional and electronic cigarettes, which promotes the development and continuation of nicotine dependence. This raises new public health concerns about the health implications of this behavior, especially that children and adolescents increasingly take up the use of e-cigarettes in some countries [8,9,10,11,12,13].

The World Health Organization underlines that both tobacco products and e-cigarettes pose risks to health, and the safest approach is not to consume either [13]. The Forum of International Respiratory Societies recommends greater research on the health effects of electronic cigarettes and surveillance of use across different countries [14].

Several studies from all over the world, including Romania, have proved that cigarette smoking is associated with several risk behaviors such as alcohol abuse and illicit drug use among different groups of young people, while the synergistic effects and augmented risks associated with concurrent use of nicotine and alcohol or other illicit drugs have been well established [15,16]. Recently, some studies, especially from North America, have focused on the relationships between e-cigarette use and risk behaviors, but few studies have been performed in Europe, and no data are available from Romania [3,17,18,19,20,21,22,23]. Further, little is known about how the intention to use e-cigarettes in the future is related to alcohol and illicit drug use patterns [18].

This study focuses on Romanian university studies from Cluj-Napoca, a big university city situated in the northwest part of Romania. It has three objectives. The first one is to assess attitudes and sources of information as well as behaviors and intentions related to e-cigarettes among Romanian university students. Second, the study aims to identify the relationship and co-occurrence of e-cigarette use and smoking and alcohol and illicit drug consumption. Third, the study assesses the relationship between intention to use e-cigarettes in the future and patterns regarding consumption of e-cigarettes, traditional cigarettes, alcohol, and illicit drugs.

## 2. Materials and Methods

### 2.1. Study Sample and Procedure for Data Collection

A cross sectional study was performed in April–May 2017 among 400 university students from the four main universities from Cluj-Napoca, a big university city situated in northwest Romania. It used an anonymous questionnaire that investigated several issues related to the lifestyles of students. 

A convenience sample of students was randomly chosen from eight dorms belonging to the four main universities of the town. This approach was used in order to have access to students from different faculties from the four universities, while contact of those students during their activities in the universities had several logistical constraints. Each university has its own dorms (the majority of dorms are only for female or male students and, if there are mixed dorms, they have separated parts for each gender group) with rooms where 2–4 students share the same room, but no information was available regarding the total number of students from each dorm. 

A convenience sample of 100 students (50 female and 50 male) living in the selected dorms were randomly chosen from each university. The selection of students was made by randomly choosing 20–25 different rooms from each dorm and asking the students living in those rooms at the time the study was performed to fill in the anonymous questionnaire. Besides gender selection, there was no pattern in the distribution of students from different faculties and years of study from the same university in shared rooms, hence one room might be shared by students from the same faculty, but with different years of study, or from different faculties belonging to the same university. The rooms where the study was performed were chosen completely randomly because no information regarding the faculty/age of participant was available, in order to help the sampling procedure.

Students were contacted directly in the dorms by a member of the research team and were informed about the voluntary participation and characteristics of the study. The refusal rate was below 6%, and the students who refused to participate were replaced with students from the same university, living in the same dorm. The participating students filled in the questionnaires, which were collected by members of the research team from each room approximately 1 h after their distribution.

The study is part of a research project that received the ethical approval (code: 120/6.03.2015; date: 6 March 2015) of the Ethic board of University of Medicine and Pharmacy from Cluj-Napoca, Romania.

### 2.2. Instrument for Data Collection

The study used an anonymous questionnaire developed for this study based on data from the literature and on previous questionnaires developed and tested in several studies from Romania [2,15,16,24]. It included several sections investigating health risk behaviors among participating students.

The present study includes information collected through the anonymous questionnaires with regard to the following issues:

- Socio-demographic characteristics (age, gender, and university);
- Opinions regarding e-cigarette use (several statements were listed and students had to declare if they agreed or not with them; possibilities for answers varying from I totally agree to I totally disagree);
- Sources of information about e-cigarette use;
- Reasons for using e-cigarettes at least once during their lifetime among those who had done so;
- Behavior related to e-cigarette use (students were asked if they had used e-cigarettes at least once during their lifetime or in the previous month);
- Intention to use e-cigarettes in the next year (students were asked if they intended to use e-cigarettes in the next year; possibilities for answers varying from definitely no to definitely yes);
- Behaviors related to smoking (students were asked if they had smoked traditional cigarettes at least once during their lifetime or in the previous month);
- Experiencing alcohol intoxication (experiencing alcohol intoxication during their lifetime or in the previous month);
- Illicit drug use (students were asked if they had used any illicit drugs during their lifetime or in the previous month).

The field of research regarding e-cigarette use has only emerged in the last few years, and no standardized questionnaire is available, with several international studies trying to assess both behavior as well as knowledge, attitudes, reasons related to trying/continuing e-cigarette use, or intention to use them in the future [17,18,19,20,21,22,23]. The assessment of smoking, illicit drug use, and experimentation with alcohol intoxication as the presence/absence of the behavior during their lifetime or in the previous month is frequently used in research studies focusing on these topics; lifetime use showing experimentation, and previous month consumption being an indicator of current use [25,26].

### 2.3. Data Analyses

Opinions, behaviors, and intention related to the use of e-cigarettes, as well as smoking, alcohol, and illicit drug use were investigated for the whole sample as well as for two distinct categories of participant-students who had tried e-cigarettes during their lifetime and those who had not done this; independent sample *t*-tests and chi^2^ tests were used to assess differences between the two groups with regard to the investigated issues.

Co-occurrences of e-cigarette use and smoking and alcohol, and illicit drug use during their lifetime and in the previous month were assessed.

Four univariate linear regression analyses were used in order to assess the association between e-cigarette use, smoking, alcohol and illicit drug use and socio-demographic characteristics. The dependent variable in each of the linear regressions was one of the four health risk behaviors (e-cigarette use, smoking, experimentation with alcohol acute intoxication and illicit drug use were coded as 0—no use/presence during lifetime, 1—use/presence during lifetime, but not in the last month, 3—use/presence in the last month), while the independent variables were the other three health risk behaviors, age, and gender. At the same time, a univariate linear regression analyze was used to assess the association between intention to use e-cigarettes in the next year (coded as (-2)—definitely no, (-1)—probably no, 0—I do not know, 1—probably yes, 2—definitely yes) and the four health risk behaviors as well as socio-demographic characteristics.

Data analysis was performed with IBM SPSS Statistics for Windows Version 20 program. Statistical significance is reported at *p* < 0.05.

## 3. Results

### 3.1. Opinions, Sources of Information, and Behavior Related to E-Cigarettes

The results show that 95.5% of the students had heard about e-cigarettes. Out of these, 43.7% had tried e-cigarettes at least once during their lifetime and 8.9% had declared using cigarettes in the previous month (one out of five students who had tried at least once during their lifetime).

Around 45% of the students considered e-cigarette less dangerous (one third of the never users versus half of the users) and helped smokers to quit smoking (41% of the never users vs. 51% of the users). Less than one third of the students considered that cigarettes were used only by smokers (almost half of the never users did not know what to say, while almost half of the users disagreed with this), a stronger disagreement being noticed among users (see Table 1).

The main sources of information regarding cigarettes were friends and the internet, followed by colleagues and point of commercial sale, statistical significant differences being noticed between users and never users, the first ones indicating the internet, friends, and colleagues as sources of information more frequently with regard to this issue.

As presented in Table 1, the reasons for experimentation with cigarettes were mainly curiosity (two thirds of users) followed by the fact that other friends also did so (one quarter). The fact that they are less dangerous than traditional cigarettes and the desire to reduce the number of traditional cigarettes was declared by 13% of users, while less than 10% had done so with the intention of quitting smoking. With regard to social influences, two thirds of the students declared they had friends who used cigarettes, and 40% declared they knew colleagues who did so; the percentage of friends and colleagues using them being higher among users.

### 3.2. Interrelationship and Co-Occurrence between E-Cigarettes Use and Smoking and Alcohol and Illicit Drug Consumption

As presented in Table 2, half of the students had smoked at least once during their lifetime and one third had smoked in the previous month. Eighty-five percent of the study sample had experimented with alcohol intoxication at least once during their lifetime and 45% had done so in the previous month. The percentage of those who had tried illicit drugs was 34%, while 9.5% had done this in the previous month. All these behaviors were statistically significantly higher among those who had tried e-cigarettes at least once during their lifetime. 

As presented in Table 3, the results of the linear regression analyses show that there is a positive correlation between e-cigarette use, smoking, experimentation with alcohol intoxication, and the use of illicit drugs, with the strongest association being between e-cigarette use and smoking as well as between experimentation with alcohol acute intoxication and smoking. Moreover, all risky behaviors were more frequent among male students than female students, while smoking was more frequent among younger ages.

Table 4 shows that only 13% of the students declared that they had not been involved in any of the four health risk behaviors (smoking, e-cigarettes, getting drunk, illicit drug use) during their lifetime, 26% had been involved only in one behavior (most frequent being acute alcohol intoxication), 14% had been involved in two behaviors (most frequently smoking and acute alcohol intoxication), 26% had been involved in three behaviors (most frequently smoking, e-cigarette use, and getting drunk), and 20% had been involved in all of the behaviors. Among users of e-cigarettes, almost all had been involved in other risky behaviors as well, around half of them had been involved in all of the investigated risky behaviors, and around one third had been involved, besides e-cigarette use, in smoking and getting drunk. 

In the previous month, 43% had not been involved in any of the risky behaviors, 26% had been involved only in one behavior (more frequently, getting drunk), 18% in two behaviors (most frequently, smoking and getting drunk), 11% in three (most frequently smoking, getting drunk, and illicit drug use, followed by smoking, getting drunk, and e-cigarette use), while less than 1% had been involved in all behaviors (see Table 4).

The sample of students who had used e-cigarette in the previous month was small, but around one third had been involved only in this behavior, while one third had also been involved in smoking and getting drunk.

### 3.3. Intention to Use E-Cigarette in the Future and Its Relationship with Other Health Risk Behaviors

One out of ten students declared their intention to use e-cigarettes in the future. As Table 3 shows, intention to use e-cigarettes in the future was higher among male students, while younger students were more convinced that they would use e-cigarettes in the future.

At the same time, intention to use e-cigarettes was associated with e-cigarette use, as well as smoking behavior, experimentation with acute alcohol intoxication, and illicit drug use; the strongest association was noticed between intention to and e-cigarette use, as well as smoking behavior.

## 4. Discussion

A study performed in 2013 among university students from Cluj-Napoca Romania showed that the prevalence of use of e-cigarettes was 25.2%, while use in the previous month was 2.3% [2]. Our study, performed in 2017, shows an increase of experimentation to 44% and that use in the previous month has risen to 8.9%. A study performed in 2017–2018 among university students from five countries situated in Central and Eastern Europe (Belarus, Lithuania, Poland, Russia, and Slovakia) showed that 43.7% of the students had used an e-cigarette at least once during their lifetime [23]. The Global Adult Tobacco Survey (GATS), performed in Romania in 2018, showed that 11.3% of the general adult population had used e-cigarettes during their lifetime, and 3.4% had done so in the previous month [27].

In the present study, similar to the Romanian study from 2013 as well as to the study performed among students from the five European countries, e-cigarette use was more frequent among male students [2,23]. The gender differences vary across countries and ages, with some studies proving a similar situation, while other did not find this [1,2,3,4,5].

The main reasons for experimentation with e-cigarettes were curiosity and the influence of other friends, similar to the Romanian study performed in 2013 as well as to other European studies [2,22]. On the other hand, one out of ten users did so to quit smoking, while, in the study from 2013, this reason was declared by one quarter of users. The thought that e-cigarette were less dangerous and an intention to decrease the number of traditional cigarettes was declared by 13% of users, which was more than the percentages found in the study from 2013 [2]. 

On the other hand, less than half of the students believed that e-cigarettes were less dangerous than tobacco cigarettes and they could help with quitting smoking, while less than one third considered that they were used only by smokers, these percentages being lower than those obtained in 2013 [2]. Moreover, students who had experimented with e-cigarette use were statistically significantly convinced that they were not used only by smokers.

Friends, colleagues, and the internet were the main sources of information; students who had tried e-cigarettes declaring to a higher extent that they had looked for information from these sources. Studies from Romania and other countries also found similar results [24,25]. Moreover, users of e-cigarettes also declared a higher percentage of friend and colleagues who used e-cigarettes, confirming the influences of peer groups in influencing opinions and behaviors, similar to other studies [18,28,29,30,31,32].

Moreover, the study from 2013 showed that less than 5% of the students declared that they will use e-cigarettes in the next year while, in our study, 10% of the participants did so [2]. In our study, this intention was stronger among male students and younger ages.

On the other hand, 36.6% of the students from our study were smokers (had smoked in the previous month), while the results of the Global Adult Tobacco Survey (GATS), performed in Romania in 2018, showed that, among the adult general population, the percentage of smokers was 30.7% [27]. Around half of the participants declared alcohol intoxication in the previous month, while almost 10% had used illicit drugs in the previous month. Another study, performed among university students from Cluj-Napoca, showed an increasing trend of illicit drug use from 1991 to 2011, with 2.5% of the female students and 6.2% of the male students declaring illicit drug use in the previous month in 2011 [33].

Previous studies performed specially in North America showed robust associations between e-cigarette use and substance-related risk behaviors, being evidence that e-cigarette use clusters with risk behaviors and appears to represent problem behavior, especially the dual use of e-cigarettes and traditional cigarettes [17,18,19,20]. Our study confirms the association between e-cigarette use, smoking, alcohol acute intoxication, and illicit drug use; students who experimented with e-cigarettes during their lifetime being statistically significantly more prone to smoking, experimenting with alcohol acute intoxication, and illicit drug use both during their lifetime as well as in the previous month. 

Moreover, the majority of the students had been involved at least once during their lifetime in at least of one of the four investigated health risk behaviors, with 60% being involved in more than one. More than half of the students had been involved in the previous month in at least one of the health risk behaviors, and 30% had been involved in more than one health risk behavior.

Intention to use e-cigarettes in the next year was also associated with e-cigarette use, smoking, and alcohol intoxication, as well as illicit drug use. Studies from other countries also showed the association between susceptibility to use e-cigarettes and consumption of other substances [18].

The results have several implications for practice. First, the data regarding e-cigarette use and the fact that both experimentation as well as its current use have increased among Romanian university students since 2013 and are more than double in comparison with their prevalence among the Romanian adult population shows the need for addressing the issue of e-cigarette use through health education programs and regulatory interventions, with a special focus on younger ages.

Second, the prevalence of smoking among students showed by the study underlines that smoking cessation programs targeting young adults should be implemented and funded properly in Romania. The use of illicit drugs also increased in comparison with studies performed between 1999 and 2011, which is of concern and requires more efforts for its prevention and reduction. Last, but not least, the co-occurrence of several licit and illicit drug use behaviors among university students calls for a comprehensive approach including education, counseling, and support services that help students to avoid and limit licit and illicit drug use as part of university policy.

The study is subject of several limitations. First, it has a cross-sectional design and the identification of causal relationships is not possible, while the study was performed in 2017 and the opinions and practices related to the use of e-cigarettes and other licit and illicit drugs among youth might have suffered changes since then. Second, it includes a convenience limited sample of 400 students living in dorms belonging to the main universities from a big town in Romania; selecting students from dorms rather than from the general student population may lead to bias and limits the generalization of the results beyond its sample. Similar to other studies in this field, the behavior of the participants was assessed based on their self reporting [25,26,27,34].

## 5. Conclusions

This is the first study from Romania and one of the few from Europe that has investigated the relationship between e-cigarettes use and the intention to use them in the future and licit and illicit drug use. It confirms the association between e-cigarette use, smoking, alcohol acute intoxication, and illicit drug use, as well as the association between intention to use e-cigarettes in the future, e-cigarette use, smoking, and experimentation with alcohol acute intoxication and illicit drug use. 

Even though the study was performed in 2017 and in the last three years, especially during the pandemic of Covid-19, several changes might have taken place with regard to e-cigarette use and consumption of different licit and illicit drugs, it still offers information regarding cigarette use among university students from Romania, a subject that has been previously investigated only by one study, performed in 2013. Moreover, the data offered by this study could be used to compare the situation regarding e-cigarette use and smoking and alcohol and illicit drug consumption among Romanian university students before and during the pandemic, as well as after the recovery period, if future studies investigate these issues.

Future research, as well as educational activities, should address the complex relationship between e-cigarettes and other substance use among Romanian youth.

## Figures and Tables

**Table 1:** Opinions, sources of information, and behavior related to e-cigarettes.

Items	Total ^a^ *n* = 382	No Experimentation ^b^ *n* = 215	Experimentation ^c^ *n* = 167
**E-cigarettes are less dangerous than tobacco cigarettes ^d^**			
Totally/partially yes (%)	44	35.7	51.6
I do not know (%)	35.2	46.5	20.6
Totally/partially no (%)	20.9	17.8	24.8
Mean (SD)	0.29 (1.07)	0.21 (0.95)	0.39 (1.20)
**E-cigarettes help for quitting smoking ^d^**			
Totally/partially yes (%)	45	41.8	51.5
I do not know (%)	27.4	36.6	15.6
Totally/partially no (%)	26.6	21.6	32.9
Mean (SD)	0.19 (1.16)	0.16 (1.01)	0.23 (1.32)
**E-cigarettes are used by smokers only ^d^**			
Totally/partially yes (%)	30.6	32.9	27.5
I do not know (%)	37.1	46.9	24.6
Totally/partially no (%)	32.4	20.2	47.9
Mean (%)	0.03 (1.05)	0.24 (0.96) *	−0.22 (1.11)
**Sources of information about e-cigarettes**			
Internet (%)	47.1	37.1 **	59.9
Commercials at sale point (%)	35.5	34.7	36.5
Newspapers (%)	5.5	4.7	6.6
Friends (%)	70	65.3 **	76
Colleagues (%)	41.3	31 **	54.5
Parents (%)	6.8	6.6	7.2
Health education activities (%)	2.6	1.9	3.6
**Use of e-cigarettes in the previous month**	8.9	-	20.4
**Reasons for using e-cigarettes at least once during lifetime**			
They are less dangerous than tobacco cigarettes (%)	-	-	13.4
To reduce the number of tobacco cigarettes (%)	-	-	13.4
To quit smoking (%)	-	-	9.3
Curiosity (%)	-	-	65.9
Other friends also have tried them (%)	-	-	26.8
**Intention to use e-cigarettes in the next year ^e^**			
Totally/partially yes (%)	10.8	2.9	21
I do not know (%)	13.8	6.2	24.6
Totally/partially no (%)	74.8	91	54.4
Mean (SD)	−1.12 (1.12)	−1.56 (0.82) *	−0.56 (1.19)
**Social influences**			
Friends use e-cigarettes (%)	72.8	63.7 **	84.4
Colleagues use e-cigarettes (%)	49.5	40.1 **	62.3
Parents use e-cigarettes (%)	3.9	3.7	4.2

^a^ The study sample consisted of students who had heard about e-cigarettes. ^b^ no experimentation with e-cigarettes during lifetime. ^c^ experimentation with e-cigarettes during lifetime. ^d^ coded as (−2)—I totally disagree, (−1)—I partially disagree, (0)—I do not know, (1)—I partially agree, (2)—I totally agree. ^e^ coded as (−2)—definitely no, (−1)—probably no, 0—I do not know, (1)—probably yes, (2)—definitely yes. * Statistical significant differences at *t*-test between student who have experimented with e-cigarettes and those who did not. ** Statistical significant differences at chi^2^ test between student who have experimented with e-cigarettes and those who did not.

**Table 2:** Health risk behaviors among university students.

	Total *n* = 382 ^a^ (%)	No Experimentation *n* = 215 ^b^ (%)	Experimentation *n* = 167 ^c^ (%)
**Smoking**			
Never	46.6	69.3 **	17.4
At least once during lifetime	53.4	30.7 **	82.7
In the previous month	36.6	16.7 **	62.3
**Alcohol acute intoxication**			
Never	16	24.8 **	4.8
At least once during lifetime	84	75.2 **	95.2
In the previous month	45.4	29 **	66.5
**Illicit drug use**			
Never	65.1	81.6 **	43.8
At least once during lifetime	34.9	18.4 **	56.2
In the previous month	9.5	2.9 **	18.1

^a^ The study sample consisted of students who had heard about e-cigarettes. ^b^ no experimentation with e-cigarettes during lifetime. ^c^ experimentation with e-cigarettes during lifetime. ** statistical significant differences at chi^2^ test between student who had experimented with e-cigarettes and those who had not.

**Table 3:** Association between health risk behaviors, socio-demographic characteristics, and mental wellbeing—results of univariate linear regression analyses.

	E-Cigarette Use ^1^	Smoking ^1^	Alcohol Acute Intoxication ^1^	Illicit Drug Use ^1^	Intention to Use E-Cigarettes ^2^
	Standardized β	Standardized β	Standardized β	Standardized β	Standardized β
**Age**	NS	−0.126 ***	NS	NS	−0.193 *
**Gender ^3^**	−0.334 *	−0.192 *	−0.421 *	−0.322 *	−0.245 *
**E-cigarette use**	-	0.445 *	0.335 *	0.374 *	0.455 *
**Smoking**	0.445 *	-	0.461 *	0.439 *	0.457 *
**Alcohol acute intoxication**	0.335 *	0.461 *	-	0.350 *	0.324 *
**Illicit drug use**	0.374 *	0.439 *	0.350 *	-	0.198 *

^1^ coded as 0—no use/presence during lifetime, 1—use/presence during lifetime, but not in the last month, 3—use/presence in the last month). ^2^ coded as (−2)—Definitely no, (−1)—Probably no, 0—I do not know, 1—Probably yes, 2—Definitely yes. ^3^ coded as 0—male, 1—female. NS—non-significant. * *p* < 0.001. *** *p* < 0.05.

**Table 4:** Co-occurrence of health risk behaviors.

	Lifetime *n* = 368%	In the Last Month *n* = 363%
**None**	**13.0**	**42.9**
**1 behavior**	**25.8**	**26.7**
Only smoking	1.4	7.7
Only e-cigarettes use	0.5	2.5
Only acute alcohol intoxication	23.9	16.2
Only illicit drug use	0.0	0.3
**2 behaviors**	**14.1**	**19.2**
Smoking and e-cigarette use	0.5	0.5
Smoking and acute alcohol intoxication	7.6	17
Smoking and illicit drug use	0.0	0.3
E-cigarette use and acute alcohol intoxication	3.3	1.1
E-cigarette use and illicit drug use	0.8	0.0
Acute alcohol intoxication and illicit drug use	1.9	0.3
**3 behaviors**	**26.5**	**10.6**
Smoking + e-cigarette use + acute alcohol intoxication	14.7	2.7
Smoking + e-cigarette use + illicit drug use	0.3	0.8
Smoking + acute alcohol intoxication + illicit drug use	8.2	6.6
E-cigarette use + acute alcohol intoxication + illicit drug use	3.3	0.5
**All behaviors**	**20.6**	**0.6**

## Data Availability

The data presented in this study are available on request from the corresponding author.
